# A novel machine-learning framework based on early embryo morphokinetics identifies a feature signature associated with blastocyst development

**DOI:** 10.1186/s13048-024-01376-6

**Published:** 2024-03-15

**Authors:** S. Canosa, N. Licheri, L. Bergandi, G. Gennarelli, C. Paschero, M. Beccuti, D. Cimadomo, G. Coticchio, L. Rienzi, C. Benedetto, F. Cordero, A. Revelli

**Affiliations:** 1 Gynecology and Obstetrics 1U, Physiopathology of Reproduction and IVF Unit, Department of Surgical Sciences, S. Anna Hospital, University of Turin, Turin, Italy; 2 IVIRMA Global Research Alliance, Livet, Turin, Italy; 3 Department of Computer Science, University di Turin, Turin, Italy; 4 Department of Oncology, University of Turin, Turin, Italy; 5 IVIRMA Global Research Alliance, Genera, Clinica Valle Giulia, Rome, Italy; 6 IVIRMA Global Research Alliance, 9.Baby, Bologna, Italy; 7 Department of Biomolecular Sciences, University of Urbino “Carlo Bo”, Urbino, Italy; 8 Gynecology and Obstetrics 2U, Department of Surgical Sciences, S. Anna Hospital, University of Turin, Turin, Italy

**Keywords:** Assisted reproduction, Blastocyst development, Artificial intelligence, Machine learning, Embryo developmental patterns

## Abstract

**Background:**

Artificial Intelligence entails the application of computer algorithms to the huge and heterogeneous amount of morphodynamic data produced by Time-Lapse Technology. In this context, Machine Learning (ML) methods were developed in order to assist embryologists with automatized and objective predictive models able to standardize human embryo assessment. In this study, we aimed at developing a novel ML-based strategy to identify relevant patterns associated with the prediction of blastocyst development stage on day 5.

**Methods:**

We retrospectively analysed the morphokinetics of 575 embryos obtained from 80 women who underwent IVF at our Unit. Embryo morphokinetics was registered using the Geri plus® time-lapse system. Overall, 30 clinical, morphological and morphokinetic variables related to women and embryos were recorded and combined. Some embryos reached the expanded blastocyst stage on day 5 (BL Group, *n* = 210), some others did not (nBL Group, *n* = 365).

**Results:**

The novel EmbryoMLSelection framework was developed following four-steps: Feature Selection, Rules Extraction, Rules Selection and Rules Evaluation. Six rules composed by a combination of 8 variables were finally selected, and provided a predictive power described by an AUC of 0.84 and an accuracy of 81%.

**Conclusions:**

We provided herein a new *feature-signature* able to identify with an high performance embryos with the best developmental competence to reach the expanded blastocyst stage on day 5. Clear and clinically relevant cut-offs were identified for each considered variable, providing an objective tool for early embryo developmental assessment.

**Supplementary Information:**

The online version contains supplementary material available at 10.1186/s13048-024-01376-6.

## Introduction

To date, an objective method to evaluate human embryo competence is still lacking, as conventional morphology is highly subjective, being performed using a wide range of grading systems affected by inter and intra-observer variability [1]. Indeed, consistency and reproducibility of embryo evaluation are still not guaranteed among different IVF laboratories [2]. In addition, standard grading only provides a momentary view of embryonic morphology at a specific time point, while developmental changes over multiple time points may provide a more robust judgement of embryo potential [3]. More than a decade ago, Time-Lapse Technology (TLT) has been introduced to perform a real-time, dynamic observation of pre-implantation embryo development. Morphokinetic events can be monitored at the exact time of occurrence, providing new insights into several steps of in vitro growth and, ultimately, suggesting objective data with potential clinical relevance [4]. TLT was claimed by some authors to improve embryo selection and IVF outcome [3–6]. However, its efficacy is still matter of debate [7–10], being mainly ascribed to unperturbed culture conditions (temperature, atmosphere), rather than to the improved identification of reliable morphokinetic biomarkers of embryo competence [8, 11]. Indeed, the real time observation of crucial events occurring during in vitro growth revealed a number of parameters that were associated to embryo development potential [4]. Looking forward, significant improvements of the technologies associated to TLT will drive a more detailed knowledge and understanding of the early developmental kinetics of human embryos [12].

Recently, Artificial Intelligence (AI) has rapidly developed in various fields, including human embryology [13, 14]. Artificial intelligence (AI) may be used as a tool to assist embryologists in daily activities (e.g. morphological selection of embryos to transfer or cryopreserve), as it is able to analyse a huge number of heterogeneous data, such as those relating to embryo development provided by TLT systems [15, 16]. Machine Learning (ML) algorithms allow the identification of the most relevant variables (also called features) included in a bulk set of data, and may be used to develop complex prediction models of embryo growth [17, 18]. Among the above-mentioned models, however, no one was able to identify clear and clinically relevant cut-offs for morphodynamic features to provide an objective tool to assist embryologists during embryo assessment.

In the present study, we aimed (i) at developing a novel ML framework using quantifiable values for both morphological and morphokinetic data of 575 in vitro-produced embryos, and (ii) at identifying which features of early embryo morphokinetics had key relevance in predicting the timely (day 5) growth to expanded blastocyst.

## Material and methods

### Patient cohorts

#### Training cohort

The study was carried out in accordance with the Declaration of Helsinki and was authorized as a retrospective observational study by the local Ethical Committee (authorization number: 0056908). A signed informed consent was obtained from all patients. The analysis included 575 embryos obtained in 80 IVF cycles performed at Physiopathology of Reproduction and IVF Unit of S. Anna Hospital (Turin, Italy) in 80 women (included only once) receiving the transfer in uterus of a single fresh blastocyst on day 5 between March 2018 and March 2020. We selected this patient population in order to have at least one viable blastocyst on day 5 to include into our analysis. These patients had mean age 35.3 ± 3.5 years (range 25–42), body mass index 24.2 ± 4.8 (range 18–25), ovarian reserve markers suggesting normal responsiveness to FSH stimulation (serum day 3 FSH < 12 IU/l, antral follicle count 8–18, anti-mullerian hormone 2.5−4 ng/ml) and were all stimulated using the GnRH-agonist “long” protocol. Patients with polycystic ovary syndrome (PCOS), ovarian endometriosis and/or unfavourable biomarkers leading to an expected poor/sub-optimal responsiveness to FSH [19] were excluded. Patients’ clinical characteristics and variables related to IVF cycle were recorded, including the total dose of exogenous gonadotropins, the number of retrieved COCs (cumulus-oocyte complexes), the ovarian sensitivity index (OSI = retrieved COCs×1000/total gonadotropin dose) [20], the fertilization, cleavage, and viable blastocyst (day 5 + day 6) formation rate.

#### Validation cohort

The performance of the *selected* rules was tested by five classification algorithms on an independent validation cohort of 81 embryos obtained from other 10 patients receiving the transfer in uterus of a single fresh blastocyst on day 5 between February and March 2020. These patients had mean age 35.5 ± 3.1 years (range 25–42), body mass index 24.6 ± 2.6 (range 18–25), ovarian reserve markers suggesting normal responsiveness to FSH stimulation (serum day 3 FSH < 12 IU/l, antral follicle count 8–18, anti-mullerian hormone 2.5−4 ng/ml) and were all stimulated using the GnRH-agonist “long” protocol.

### Controlled ovarian stimulation and IVF procedures

The gonadotropin-releasing hormone (GnRH)-agonist “long” protocol with recombinant FSH (Gonal-F®, Merck, Germany) at individually tailored daily dose (100–300 IU s.c.) was always used to carry out controlled ovarian stimulation (COS). However, the observed results are likely to be applyable also to GnRH-antagonist cycles, as a previous study demonstrated that the OSI is not significantly affected by the type of pituitary suppression, being highly consistent when the same patient undergoes GnRH-agonist “long” protocol and then GnRH-antagonist protocol or vice-versa [21]. Circulating estradiol (E2) assessment and transvaginal ultrasound (US) examination were performed every second day from stimulation day 7 in order to monitor follicular growth. The FSH dose was adjusted accordingly. A single subcutaneous injection of 10,000 IU hCG (Gonasi HP, IBSA, Switzerland) was administered to trigger ovulation when at least two follicles reached 18 mm mean diameter, with appropriate E2 levels. US-guided oocyte retrieval (OPU) was performed 35–37 h after hCG trigger under local anaesthesia (paracervical block).

Sperm concentration, motility, and morphology were assessed according to the World Health Organization guidelines [22]. Raw semen was assessed for both progressive and non-progressive motility. After density gradient centrifugation, sperm motility was expressed in terms of concentration of activated cells with progressive motility.

At OPU, the aspirated follicular fluids were immediately observed under a stereomicroscope, cumulus-oocyte complexes (COCs) were washed in buffered medium (Flushing medium, Cook Ltd., Ireland) and oocytes were inseminated within 4 h using either conventional IVF (34%, 27/80 cycles) or ICSI (66%, 53/80 cycles), according to semen quality. When performing ICSI, 2 h after OPU oocytes and cumulus cells (CCs) were separated from each COC by gently pipetting in a 40-µl HEPES buffered medium containing 80 IU/ml hyaluronidase (Synvitro Hyadase, Origio Medicult, Denmark) [23]. Normal fertilization was confirmed when two pronuclei (2PN) and the extrusion of the second polar body were observed within 16–18 h after insemination.

### Annotation of embryo morphokinetics

A total of 575 embryos were included into our analysis, obtained either with conventional IVF (*n* = 216) or ICSI (*n* = 359). Embryos were cultured in the Geri plus® TLS (Genea Biomedx, Australia), that is equipped with an integrated embryo monitoring system to observe one zygote/microwell, as previously described [24]. The dish format allowed the observation of each embryo individually at 11 different focal planes, even if all embryos shared a common 80-µl medium drop. Up to day 3, embryos were cultured in pre-equilibrated Cleavage medium (Cook, Ireland) overlaid with mineral oil; then, a change of medium was performed, and the new medium (Blastocyst medium, Cook) was kept until the blastocyst stage. Bright-field images were captured by Geri plus® system every 5 min from the time of fertilization until day 5, when embryo transfer (ET), cryopreservation or discharge occurred.

Embryo morphological evaluation was first performed by one single senior embryologist on day 2 using the Integrated Morphology Cleavage Score (IMCS) [25], and then repeated on day 5 according to standardized criteria [1]. Notably, IMCS was based on the evidence of implantation and clinical pregnancy after double ET on day 2, and was incorporated into a complex prediction model for IVF outcome, recently shown to predict live birth with a remarkably good precision [26]. However, prospective application of that model proved that it was highly effective as a means of embryo selection to reduce twin implantation rates (from 28 to 2%) for both SET and DET [27]. Furthermore, all videos collected by Geri plus® were analysed by the same senior embryologist, and based on ESHRE recommendations [4] the following morphokinetic parameters (times) were manually annotated on all generated embryos, although affected by unavoidable intra-operator variability: pronuclear appearance (tPNa), pronuclear fading (tPNf), completion of cleavage to two, three, four, and eight cells (t2, t3, t4, and t8, respectively), time intervals tPNf-tPNa, t2-tPNf, t3-t2, t4-t3, t4-t2, and t8-t4. Day 5 blastocyst formation was assessed at the same time interval (116 ± 2 h post insemination) for all embryos. Embryos reaching the expanded blastocyst stage on day 5 (score 3 according to [1] were included in the Blastocyst Group (BL Group, *n* = 210), whereas those arrested or progressed to a stage earlier than expanded blastocyst were included in the Not-expanded Blastocyst Group (nBL Group, *n* = 365), as previously described [28]. We decided to consider blastocyst expansion as the main criteria to identify BL or non-BL group, irrespectively of ICM and TE morphology, because it was recently confirmed that although embryologists may adopt the same grading scheme their agreement is limited, especially when the morphological quality is low [29–31].

### Training dataset preparation

A total of 30 variables among those currently recorded during clinical routine were considered for each embryo and divided into three categories: (i) woman-related (*n* = 6) describing patients clinical characteristics: age, BMI, day 3 FSH, AMH, antral follicle count (AFC), years of infertility; (ii) COS-related (*n* = 10), describing the clinical characteristics and outcomes of ovarian stimulation: total exogenous FSH, peak E2, OSI, number of retrieved oocytes, number of mature oocytes, maturation rate, number of fertilized oocytes, fertilization rate, number of cleaved embryos, cleavage rate; (iii) embryo-related (*n* = 14), describing embryo morphology and mophokinetics: insemination technique, IMCS score on day 2, tPNa, tPNf, t2, t3, t4, t8, tPNf-tPNa, t2-tPNf, t3-t2, t4-t3, t4-t2, t8-t4. Specifically, all the considered variables (*n* = 30) were evaluated for each embryo obtained by ICSI (*n* = 359), whereas for embryos obtained by conventional insemination (*n* = 216), tPNa and tPNf-tPNa were not annotated, as in this case normal fertilization was checked only after cumulus cells removal. Overall, this dataset composed the Training cohort used for ML framework development.

### Development of the EmbryoMLSelection framework

The generation process of the novel EmbryoMLSelection framework was composed of four modules: Features Selection, Rules Extraction, Rules Selection and Rules Evaluation (Supplementary Fig. 1), in order to easily analyze embryo data and build a model able to distinguish between two groups of embryos: one characterized by a high likelihood of reaching the expanded blastocyst stage on day 5 (referred to as the BL group) and the other with a low chance (referred to as the nBL group). The initial component of EmbryoMLSelection is Feature Selection, which involves identifying the most informative features for the task while eliminating noisy, non-informative, irrelevant, and redundant features. The resulting list of features is then inputted into the subsequent step, called Rule Extraction, focusing on exploring rule-based models. Subsequently, a Rule Selection process is performed to eliminate unpredictable or redundant rules. In detail, each rule was evaluated in terms of the number of true and false positive/negative samples classified; these values were used to compute the Matthews Correlation Coefficient MCC, a measure of the quality of the binary classification prediction specifically for the imbalance dataset. MCC reflects how well the rules were able to distinguish between the class: specifically, MCC was used to identify those rules having a limited overlap in terms of both the features considered and the samples correctly classified, leading to an increase in the number of samples correctly classified. The MCC values must be interpreted as a correlation index (i.e. positive/negative values indicates direct/inverse relationship, while 0 implies random correlation). The Rules Evaluation phase is then implemented to assess the model’s performance by measuring how accurately its predictions align with the observed data. A graphic formalism was adopted to visualize the functional dependencies among variables.

The machine learning methods devoted to the classification task are generally grouped into four overlapping categories: (i) the geometric models represent instances as points in a high-dimensional Euclidean space and exploit spatial concepts (e.g. distances, lines, planes) to make decisions. In this category, we have implemented two algorithms support vector machines (SVM) and k-nearest neighbors (kNN). (ii) The probabilistic models assume there is an underlying random process modeling the relationship between the data and the target variable (i.e. BL or nBL groups), and try to model it using probability distributions. Logistic regression estimates the probability of a binary outcome by fitting a logistic function to the linear combination of input features, while Naive Bayes is based on Bayes’ theorem with the naive assumption of feature independence. (iii) The rule-based models partition the instance space into instance space segments through a set of logical rules, using either a list or a tree-based structure. Decision trees are a popular rule-based model for classification and regression tasks; in this category, we have implemented the Random Forest algorithm. Finally, (iv) ensemble methods are related to a set of techniques wherein a target function is learned by training multiple individual learners and then consolidating their predictions. Gradient boosting and Ada Boosting are the two methods implemented in this category. The AUC and accuracy values reported in the paper are the highest values obtained from each of the five classification algorithms.

The analysis workflow was implemented using Python 3 programming language [32]. detailed description of the modules and the methodology adopted is provided in the supplemental Material and Methods. EmbryoMLSelection framework was registered in the Docker image, available at https://hub.docker.com/r/qbioturin/embryo_ml_workflow. The web page contains the list of requirements and the command line to run the code. An example of how to run the workflow using the Embryos input data and the complete list of the parameters is provided in order to ensure both functional and computational reproducibility of the experiments.

### Statistical analysis

Principal Component Analysis (PCA) was executed using the scikit-learn library in Python. The heatmap, illustrating R coefficients derived from Pearson correlation between feature pairs, was generated using the Python Data Analysis Library called pandas. The arrangement of rows and columns is determined by grouping features based on their types. Prior to PCA and correlation analysis, feature values are normalized as z-scores.

## Results

### Correlation between the considered variables

**Fig. 1:**
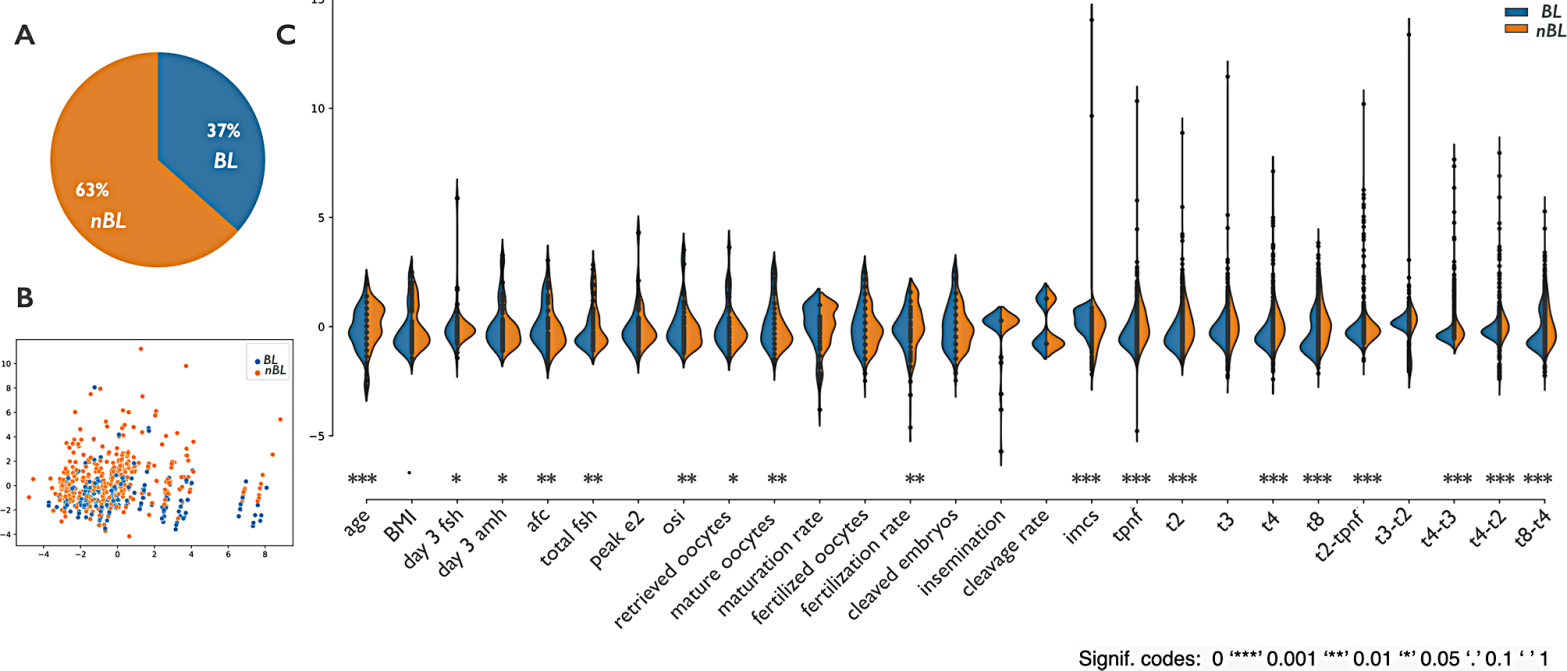
(**A**) Pie chart showing the distribution of the 575 embryos: embryos progressed to the expanded blastocyst stage on day 5 (BL, blue) or not (nBL, orange). (**B**) Scatter plot obtained from a dimensionality reduction technique (principal component analysis) considering all 575 embryos and all variables. The color of the embryos identifies those grown to the expanded blastocyst stage on day 5 (BL, blue) or not (nBL, orange). (**C**) Split violin plots of the distribution of the z-score of the value of all features distinguishing embryos grown to the expanded blastocyst stage on day 5 (BL, blue) or not (nBL, orange). Each violin plot is divided in half allowing for a clear observation of differences in the distribution of values between the BL and nBL groups. The p-values expressed by a significance code are computed by a Kruskal-Wallis rank sum test

Supplementary Table 1 summarizes the clinical characteristics of the 80 patients included into the Training dataset and the outcome of their IVF cycles. Overall, 575 embryos were obtained, of which 210 (36.5%) progressed to the expanded blastocyst stage on day 5 (BL group) whereas 365 (63.5%) did not (nBL Group) (Fig. 1A).

At first, all embryos were considered together for a preliminary analysis aimed at identifying a correlation cluster between the considered variables and embryo development where we applied the Principal Component Analysis (PCA) to generate a labelled scatter plot according to the clustering category of the embryos (BL or nBL). Although there was not a clear separation of the two embryo populations (Fig. 1B), different density curves could be noted after comparing the range of values of BL with those of nBL, as shown in Fig. 1C (see also Supplementary Table 2).

**Fig. 2:**
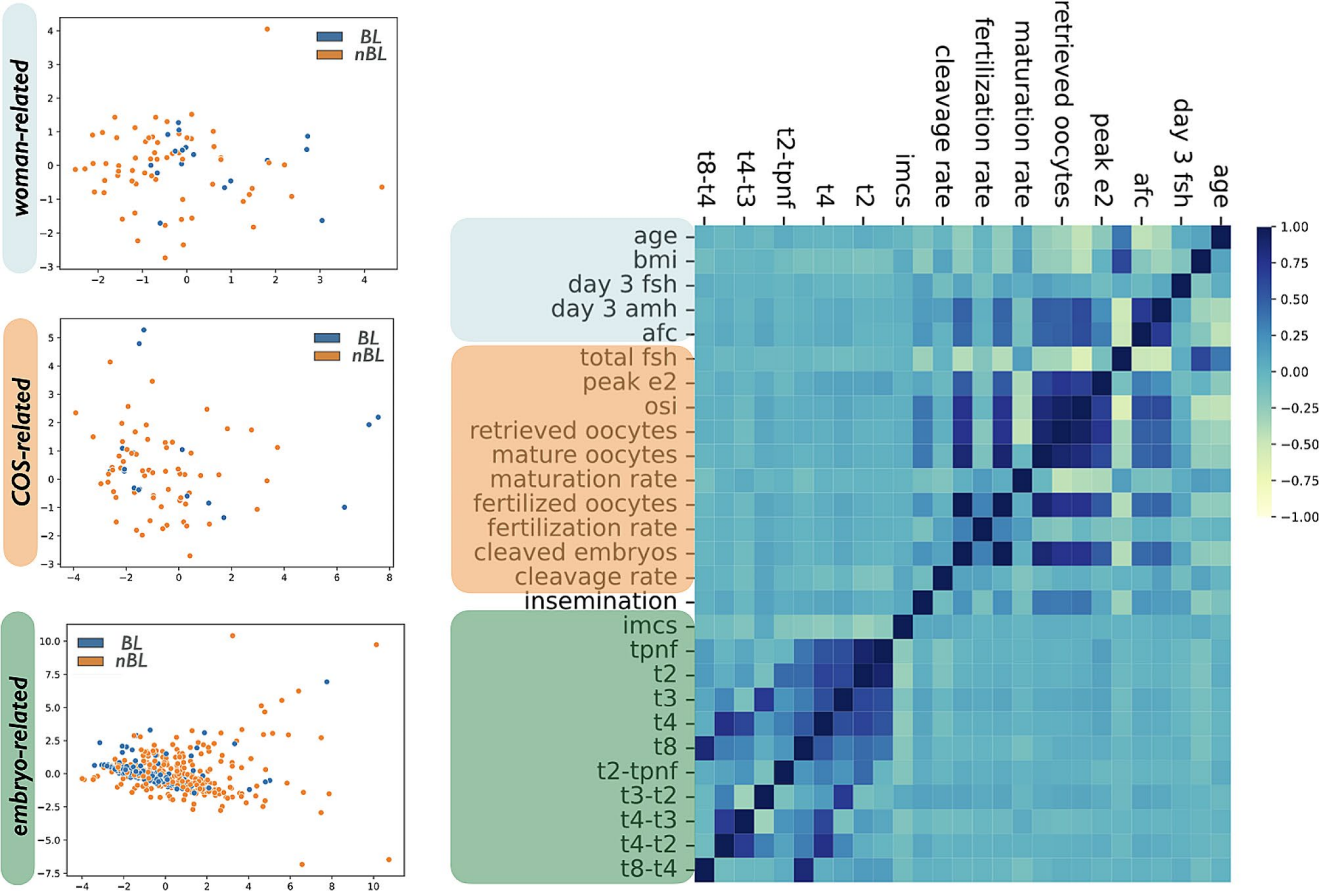
Heatmap plot showing the Pearson correlation value among all variables (right panel of the figure). Scatter plots computed through a dimensionality reduction technique (principal component analysis) for each set of variables, independently (leftpanel)

The inter-variables correlation analysis revealed two major clusters, the first between woman-related and COS-related variables, the second within embryo-related variables, as depicted in the heat map (Fig. 2). Interestingly, the insemination technique showed either weak or no correlation with the other variables (Fig. 2). In addition, a clear cluster was not detectable considering each set of variables to discriminate the BL and nBL embryo groups by scatter plots (Fig. 2), thus concluding that our preliminary analysis was not enough to identify predictive variables of embryo development.

### Selection of rules associated with blastocyst development

**Fig. 3:**
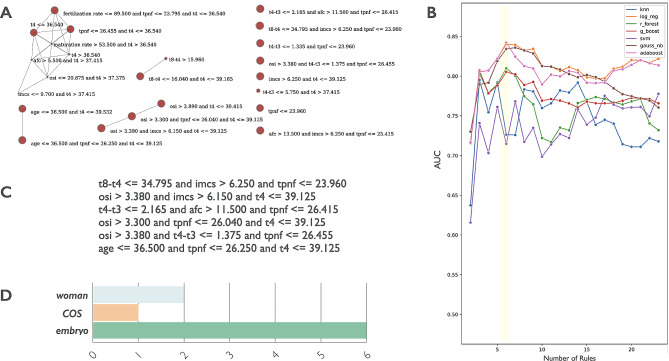
(**A**) Correlation graph of the selected rules. Vertices represent the rules and arcs are reported only for a correlation value > 0.8 (computed by Matthews Correlation Coefficient (MCC). (**B**) Line plot representing the ability of different combinations of classifiers to classify the expanded or not expanded blastocyst stage. Each dot corresponds to the AUC computed using a different number of rules in input. (**C**) Set of 6 rules of the *feature-signature* with (**D**) their composition in terms of variable category

To pursue our aim to define a specific feature signature, the first phase of EmbryoMLSelection framework was applied to identify the most predictive variables. This process exploits multiple selection algorithms (e.g. filter and embedded methods) to explore the ideal set composed by balanced cut-off between the number of features and the power of their association with embryo development. The selection strategy was performed on the Training cohort using the 70% (*n* = 403) of the total number of embryos defined as training dataset. The identified set of features was tested on a classification task using stratified 10-fold cross validation repeated 100 times against the test set composed of the remaining 30% (*n* = 172) of the embryos of the Training cohort. A total number of 12 variables (OSI, Maturation rate, Fertilization rate, Cleavage rate, Age, Day 3 FSH, AFC, IMCS, tPNf, t4, t4-t3, t8-t4) composed a preliminary set providing the highest area under the curve (AUC = 0.74 obtained by the gradient boosting classification algorithm). In a second phase, these variables were managed to define the set of rules (combination of variables) able to discriminate embryos of BL and nBL groups. The Rules extraction module was then applied to generate 71 rules from the training set. To explore the association of the *extracted* rules within each other and with blastocyst development, the Rule Selection module was applied to implement the Matthews Correlation Coefficient (MCC). MCC was used to remove the unpredictive or redundant rules, leading to a new set of 23 selected rules. Figure 3A is the correlation graph obtained from the visualization module, where the rules were represented as nodes whose size is correlated with the relevance of the primary outcome (blastocyst development), while the edge between two nodes represented a correlation value for those rules higher than 0.8 (computed by Matthews Correlation Coefficient (MCC). It is interesting to note that 8 out of 23 rules were isolated vertices (Fig. 3A, red nodes on the right) highlighting their low (i.e. minor than 0.8) functional dependency from the others rules but, at the same time, their high relevance with the primary outcome (the size of the node was generally large).

We defined a *feature-signature* composed of 6 out of the 23 selected as the rule combination with the highest discrimination power. This evaluation was performed in terms of classification performance expressed as AUC value. Specifically, Fig. 3B shows the line plot reporting the AUC values (y-axis) computed by the seven classification algorithms for the classification of the expansion or not of the blastocyst, considering a given number of features (x-axis). The maximum value of the AUC (0.842) was reached by the AdaBoosting classifier considering 6 rules (yellow vertical ribbon); all coefficients computed for all the seven classifiers are reported in Supplementary Table 3. Figure 3C reported the 6 rules and their feature composition based on: one COS-related feature (OSI), two woman-related features (i.e. Age and AFC), and five embryo-related variables (i.e. tpnf, t4, t4-t3, t8-t4, imcs) (Fig. 3D).

### Validation of the selected rules on an independent dataset

The performance of the 6 *selected* rules was tested by five classification algorithms on an independent validation cohort of 81 embryos obtained from other 10 patients with clinical characteristics comparable to the women included in the study (Supplementary Table 1). We confirmed that the rules composting the *feature-signature* showed a predictive performance with AUC = 0.842 and accuracy of 0.81. For the sake of convenience, the EmbryoMLSelection framework was also applied in a setting in which the variables were not previously selected. Specifically, all variables were used together in order to obtain at first 131 extracted rules, showing a high functional dependency highlighted by the high number of arcs (Supplementary Fig. 2A). From a subset of 30 selected rules, 17 were finally derived based on the AUC in order to choose the best combination of rules with the highest discrimination power (Supplementary Fig. 2B). However, in the validation cohort these rules reached lower values in terms of both AUC (0.79) and accuracy (0.70) with respect to the previously identified *feature signature.* Finally, when considering only the embryo-related variables, 146 rules were extracted with high functional dependency (Supplementary Fig. 3A). A subset of 30 selected rules allowed to identify, in the validation cohort, a final number of 21 rules reaching an AUC value = 0.74 and an accuracy value = 0.68 (Supplementary Fig. 3B). This performance confirmed that embryo-related variables alone are not enough to accurately describe blastocyst development, and that it is necessary to consider the overall set of variables while designing the framework.

## Discussion

Artificial Intelligence is one of the most promising, objective methodologies aimed at standardizing embryo assessment in human IVF. An intriguing application of AI within the IVF laboratory is providing new knowledge on cellular profiles regulating embryo in vitro growth and embryo competence [14]. In particular, the use of explainable methods, as well as the rules-based models, makes the classification problem (i.e., the association of a particular object to a class considering a subset of features describing the object) understandable, transparent, and interpretable.

The introduction of TLT offers the possibility to obtain a vast bulk of data on the kinetic of human embryo growth, producing a much more detailed timeline of dynamic events as well as showing previously unrecognizable phenomena [33].

Exploring the ability of all features, clinical, TLT and patient’s characteristics to distinguish BL versus nBL embryos, become clear that dimensionality reduction methods, as well as Principal Component Analysis, ineffective in discerning between the embryos.

In the present study, we describe the performance of the novel EmbryoMLSelection framework in identifying a set of rules associated to a timely embryo development to the expanded blastocyst stage on day 5, called *feature-signature*. The rationale behind a two-step process of features selection, rule extraction and rule selection from a large number of variables/embryo (*n* = 30) was the identification of a set of rules (from an initial number of 71 to the final 6) identifying an *feature-signature* composed of relevant features (*n* = 8), describing cleavage stage embryos able to timely (within day 5) progress to the expanded blastocyst stage. As lower implantation and clinical pregnancy rates were reported in case of transfer of slow-growing blastocysts vs. fully expanded day 5 blastocysts [34, 35], probably due both to a poor embryo competence and to the loss of embryonic-endometrial synchrony [36], we considered the fully expanded blastocyst on day 5 as the optimal development stage, that confers the highest probability of embryo implantation. In fact, the number of days of blastocyst development represents the developmental potential of a blastocyst [37] and affects the outcome of transfer [38]. In addition, the developmental potential of Day 5, 6 and 7 blastocysts decreases gradually with the extension of culture time [39]. Therefore, the conventional practice in the laboratory is to select blastocysts for transfer, biopsy or cryopreservation, starting from expanded day 5 blastocysts. In our dataset, 36.5% progressed to the expanded blastocyst stage on day 5 (BL group defined according to the score 3 provided by the Istanbul Consensus) whereas 63.5% did not (nBL Group). This data should be discussed in relation to the overall blastocyst formation rate of 53.8% observed in the enrolled patients. In fact, according to the Vienna Consensus [40], we considered also blastocysts with expansion score of 2 in the KPI “blastocyst formation rate”, excluded from the BL group of our study. For this reason, we believe we can exclude any selection bias from the patients population ensuring an adequately powered analysis during the framework development. Embryo selection models developed using morphokinetic parameters were previously shown to predict blastocyst development [41, 42]. Furthermore, the application of machine-learning technology provided an algorithm able to predict clinical pregnancy and live birth rate by analysing embryo morphokinetics [43]. Giscard d’Estaing [18] used a machine-learning system in order to build up a score for blastocyst formation with a prediction power having AUC = 0.634. In another study, the prediction accuracy of embryo assessment performed by experienced embryologists with morphokinetic grading methods added to conventional static morphology was shown to range between 60% and 70%, with AUC = 0.63–0.70 [44]. In a previous study, the efficacy of six in-house embryo-selection algorithms (ESAs) was investigated in a set of known implantation embryos [45]. Interestingly, although the primary endpoint considered and the nomenclature adopted were different, we both observed that tPNf, s2 (i.e. t4-t3) and cc3 (i.e. t8-t4) were associated to embryo developmental and reproductive competence. Since their results highlighted that ESAs are usually specific to the patient, treatment, and environment, we agree that currently available algorithms should be carefully validated before consider clinical applicability and, more importantly, they risk to lose their diagnostic value when externally applied. Herein, we provide evidence that the novel EmbryoMLSelection framework allowed to perform a more precise evaluation of embryo dynamic growth with a performance described by AUC = 0.84 and accuracy of 81%. Notably, the Rules Selection step ensured such an increased performance providing a concomitant reduction of the rules and variables used (*n* = 6 and 8, respectively). Importantly, the EmbryoMLSelection framework developed here was registered in the Docker image and therefore its application is globally accessible online. Of note, the rules associated with the ability of reaching the stage of expanded blastocyst on day 5 include early embryo-related variables, such as embryo morphological score on day 2, and some cytokinesis times occurring in the first three days of development (tPNf, t4, t4-t3, t8-t4). So far, only one study coupled TLT annotation with morphological embryo assessment performed with the evidence-based score named IMCS [46]. According to our results, good quality embryos having static morphological score > 6.0 on day 2 are more likely to reach the expanded blastocyst stage on day 5. In addition, the relevance of timings describing early embryo development is confirmed by previous studies reporting that a timely blastocyst development on day 5 can be predicted looking at the first three days of development [28, 47]. Moreover, morphokinetic data of cleavage stage embryos were found to be associated to both blastulation rate and blastocyst quality [48]. Indeed, embryos with quicker cleavage time from the 2-cells to the 8-cells stage have the highest potential to timely become blastocyst with good morphological score, and with the ability to expand and implant [49, 50]. In this context, the pivotal clinical significance of our framework would be to indicate on day 3 which embryos are more likely to develop into viable blastocysts, giving the potential advantage to select the most competent embryos on day 3 without the need to extend culture till day 5, thus saving time and resources.

Moreover, other clinical variables, such as age, AFC and OSI, were associated to the timely progression to the blastocysts stage.

Indeed, female age defined as advanced (AMA; >35 year) was extensively associated with a decline in oocyte yield, fertilization, and overall oocyte/embryo developmental competence, mainly due to an increased incidence of aneuploidies and a decreased mitochondrial activity [51, 52]. Studies reporting embryo morphokinetics from the fertilization to the pre-implantation period in women of AMA remain limited; in our dataset, 44 (55%) of cycles were performed in patients with AMA and a total of 303 (53%) embryos were included in our analysis suggesting a link between morphokinetic pattern and maternal age. Maternal age seems to have a relevant impact on the regulation of cell polarity during compaction, as well as on blastocoel cavity expansion, suggesting that AMA may affect embryo competence irrespective of the well-known consequences of oocyte meiotic errors [53]. On the other hand, AFC and OSI are markers of ovarian reserve and responsiveness to COS, and are associated not only with female age, but also with circulating AMH levels, oocyte yield and, ultimately, clinical pregnancy [54, 55].

Interestingly enough, the insemination technique (conventional IVF or ICSI) was not included as relevant variable in the selected rules, confirming previous evidence showing only minor morphokinetic differences between the two procedures [56].

Importantly, the rules proposed in this study are presented as a signature rather than a machine learning model. Despite the AdaBoosting algorithm had the highest AUC value in discriminating between BL and nBL and provided a model comprising rule-to-weight associations which influence each rule’s contribution to the final prediction, our focus was primarily to identify the rules rather than their weight.

The present study has the following limitations: (i) only couples undergoing single blastocyst transfer were considered in this study; (ii) the overall number of considered embryos was limited (*n* = 575) but it constituted the entire time-lapse database available in our centre; (ii) embryo developmental timings were manually annotated, with unavoidable intra-operator variability [57]; (iv) only blastocyst expansion was considered irrespectively of ICM and TE morphology, but a timely blastocyst formation has a limited association coefficient with embryo ploidy and implantation chance [58]. As a consequence, we recognise that our analysis was performed on a restricted patient population with a limited sample size, thus making the current findings less generalizable in different clinical and laboratory settings.

## Conclusions

To summarize, we identified specific rules composed of a combination of demographic, morphological and morphokinetc variables, that can represent a *feature signature* significantly associated with the likelihood to progress to the expanded blastocyst stage on day 5. We also identified for the first time clear and clinically relevant cut-offs for each considered variable, providing new insights on the critical range of values affecting embryo developmental competence.

In our opinion, it is likely that AI will be soon widely used in IVF labs, as it may be of high value in Lab automatization [59, 60]. Further studies are needed to prospectively validate our framework in a clinical setting, aimed at assessing whether it may provide a more objective, accurate, rapid and standardized tool for the assessment of embryo development.

### Electronic supplementary material

Below is the link to the electronic supplementary material.

Supplementary Material 1

Supplementary Material 2

Supplementary Material 3

Supplementary Material 4

## Data Availability

The study data are available on reasonable request.
