# Supplementary figures and images for: A novel machine-learning framework based on early embryo morphokinetics identifies a feature signature associated with blastocyst development
Source: J Ovarian Res. 2024 Mar 15;17:63. doi: 10.1186/s13048-024-01376-6 (PMC10941455; doi:10.1186/s13048-024-01376-6)

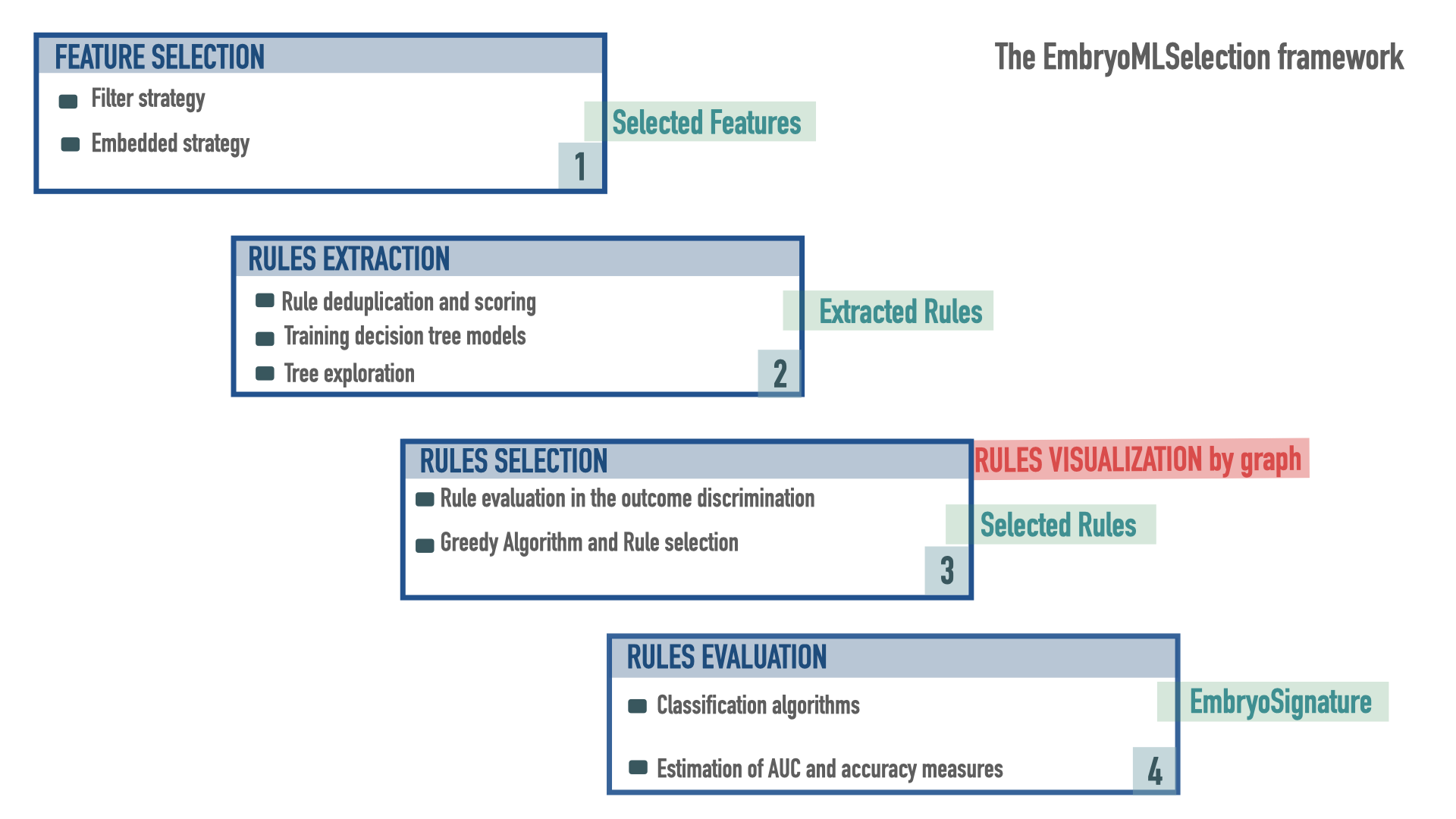

Supplement: Supplementary file 1 — Supplementary Material 1 [file 13048_2024_1376_MOESM1_ESM.tiff]

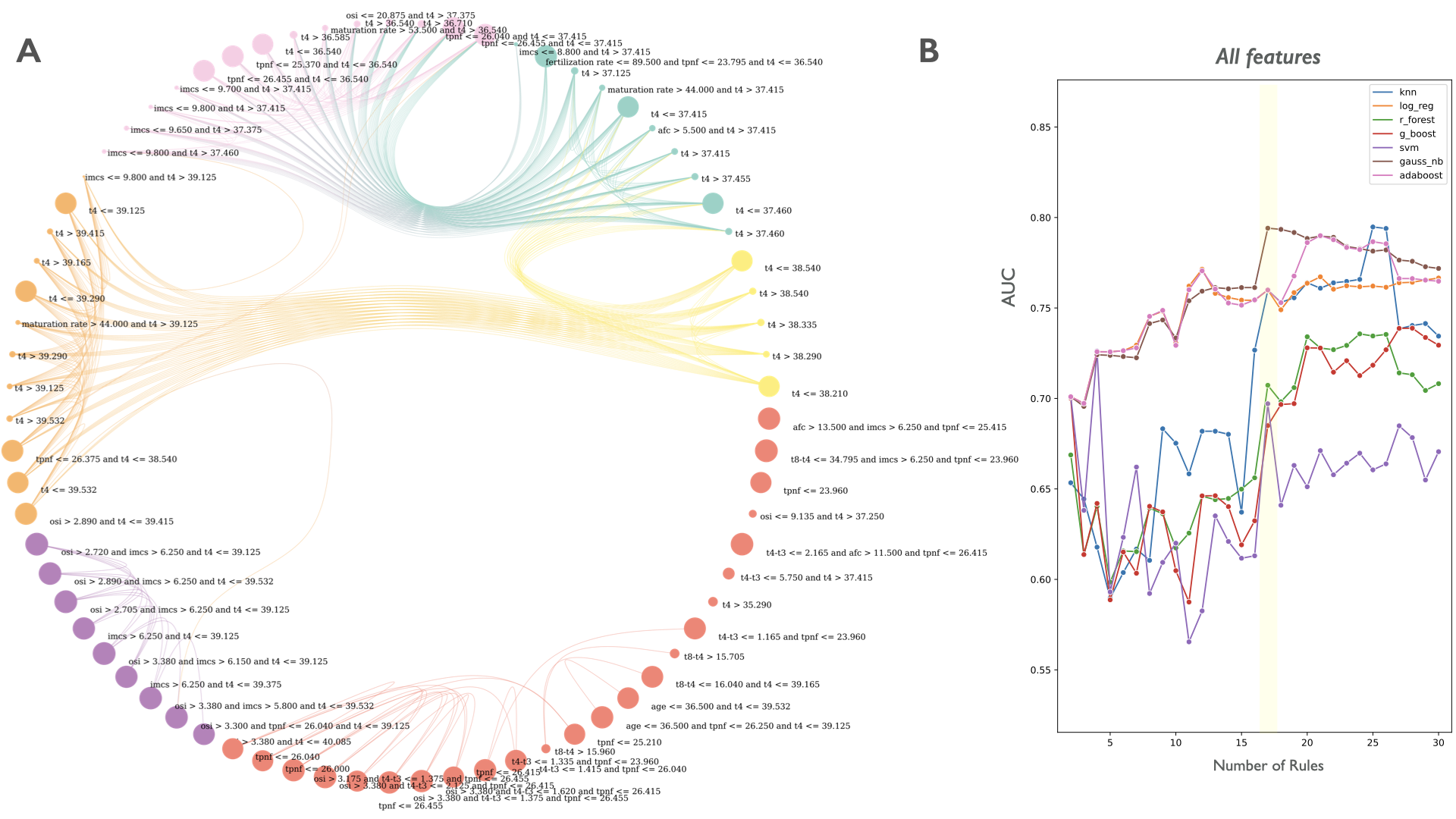

Supplement: Supplementary file 2 — Supplementary Material 2 [file 13048_2024_1376_MOESM2_ESM.tiff]

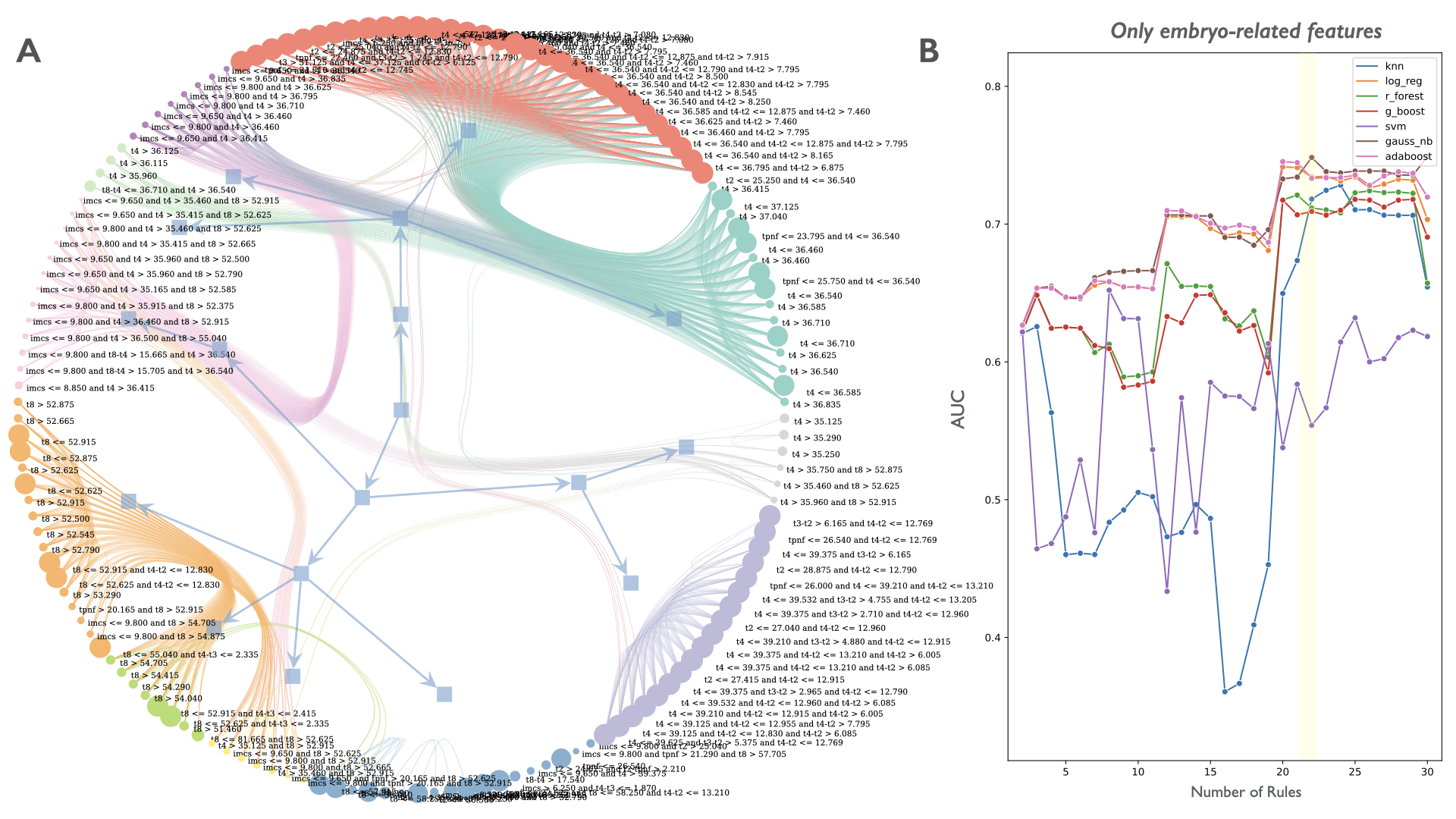

Supplement: Supplementary file 3 — Supplementary Material 3 [file 13048_2024_1376_MOESM3_ESM.tiff]
