# Supplementary material for: A novel machine-learning framework based on early embryo morphokinetics identifies a feature signature associated with blastocyst development
Source: J Ovarian Res. 2024 Mar 15;17:63. doi: 10.1186/s13048-024-01376-6 (PMC10941455; doi:10.1186/s13048-024-01376-6)
Supplement: Supplementary file 4 — Supplementary Material 4 [file 13048_2024_1376_MOESM4_ESM.docx]

**SUPPLEMENTAL MATERIAL AND METHODS**

**Development of EmbryoMLSelection framework**

EmbryoMLSelection framework is composed of four modules: Features Selection, Rules Extraction, Rules Selection, and Rules Evaluation (Supplementary Figure 1). A graphic formalism was adopted to visualize the functional dependencies among variables.

i) Feature Selection. To identify the k-best features from a given set of variables, multiple selection criteria are available. In [1] we implemented and used a feature selection phase to identify the most relevant and nonredundant features in the distinction of the samples between groups of interest. This module is based on the same filter methods proposed in [1], specifically, the analysis of variance Ftest and mutual information were adopted as filter methods, whereas the embedded methods were based on logistic regression and random forest. Specifically, the module for ensemble feature selection employs data and function perturbation techniques with the aim of identifying robust and predictive feature sets. In addition to the input data parameters, the Feature selection module takes as input: (i) nfmin and nfmax, which define the range [nfmin, nfmax] of the number of features to take into consideration; (ii) the data perturbation parameters, namely k and t, which are used as arguments for a repeated stratified k-fold cross validation; (iii) the function perturbation parameters, which is a non-empty list of feature importance rankers, called selectors, to predict crucial features from a given set of data. The feature selection phase explores the feature set search space by varying the feature set length in the interval from nfmin to nfmax. Considering a portion of data, a selector s and the number of features to select n, the algorithm exploits the selector to predict the n most important features. Whenever a feature set is identified, the number of times it has been identified is incremented, in order to take into account the number of times each set has been identified to evaluate the stability of each set. The performance evaluation phase is implemented once the feature selection is terminated. The candidate set is evaluated in a cross-validation setting in order to obtain a set of metrics for each feature set. Results of the evaluation are exploited to rank the candidate feature sets and to provide as output the ones showing the highest classification performances on the provided test sets.

ii) Rule Extraction. The rule extraction phase, also called rule mining, was performed by training decision tree models on the training set (database containing demographic and ovarian stimulation data of the women, as well as morphological and morphokinetic data of their 575 cultured embryos) in a stratified cross-validation setting. All the paths starting from the root of a fitted tree to one of the leaves were collected in order to build the logical rule set R. Let p be a path from the root up to leaf at depth n: then, the resulting logical rule is the conjunction of n terms, such that each term is a feature constrained with a numerical threshold. For example, let t8 <= 74.02 and bmi > 19 be a logical rule including two variables (i.e. bmi and t8). The set R of r extracted rules is then evaluated on the whole training set resulting in an N x r boolean matrix M such that each row i belongs to the i-th sample in the training set, and column j is the evaluation of the j-th rule from the extracted ones. The element i,j in matrix M indicates whether the j-th rule is either true or false in the i-th sample. The Matthews Correlation Coefficient (MCC), a measure of association between binary variables whose value ranges from -1 to +1, was estimated between each rule and the outcome variable [2]. Subsequently, rules were deduplicated: they were grouped by the variable(s) appearing in the rule, and only the rule with the highest MCC was considered. Finally, the rules were ranked based on the absolute MCC value to remove those associated with poor accuracy. To explore whether the selected rules can discriminate between positive and negative examples, the K-modes algorithm [2] was applied. K-modes is an unsupervised clustering specific for categorical variables. Silhouettes and the elbow plot were exploited to identify the appropriate number of clusters. Clustering was the starting point for the second step of rule selection to identify a subset of rules having a cluster-specific distribution. Finally, such a rule set was reduced to keep a set of pairwise independent rules which are all associated with the outcome (i.e. BL or nBL Group).

iii) Rules Selection. K-modes up to N clusters were applied on the data aiming to identify rule patterns able to split the data into ideally pure clusters. Once samples had been assigned to the cluster, a cluster-specific variable correlation graph was calculated considering samples in the i-th cluster as an independent dataset. Then, cluster-specific graphs were unified into a single graph by reweighting vertices and edges based on sizes of clusters. Let Ni be the number of samples falling in the i-th cluster and let N be the total number of samples. Then, the i-th cluster contributed with a weight wci=Ni/N. From such a graph, the vertices selected are those having: (i) a number of edges below the threshold defined as the median number of neighbours + one standard deviation; and (ii) a weight above the median vertex weight + one standard deviation.

iv) Rules Evaluation. Five classification algorithms (described below) were used to evaluate the rule set extracted. The dataset was partitioned into training and test sets with a 70/30 proportion. Given a set of variables, the classification algorithm evaluation was performed by fitting all the algorithms in a 10-fold stratified cross-validation procedure, getting the average performances of the algorithm on the test set and the independent validation set.

**Rules visualization by correlation graph**

A graphic formalism was adopted to visualize the functional dependencies among variables. A double-weighted graph G=(V, E), namely a graph in which both edges and vertices are weighted, was used for this purpose; in it, the number of vertices is equal to the number of variables considered. A vertex vi was weighted as wi which represents feature fi’s relevance, estimated using the MCC between the values of fi and the outcome. Let us define a correlation threshold ct ≥ 0. There exist an edge between two vertices vi and vj if MCC(fi, fj) above the threshold ct. The presence of an edge ei,j indicates that fi and fj are functional dependent over the threshold selected. From a graphical point of view, the sizes of the vertices are proportional to vertices’ weights, wi, while the thicknesses of the edge are equivalent to magnitude of MCC (fi, fj).

**Classification algorithms**

Seven machine-learning algorithms classifying the samples were exploited in order to identify those performing better with the available data: (i) Support vector machines (SVM) algorithm represents each sample as a point in a high-dimensional Euclidean space and learns a decision boundary to separate positive from negative samples [4]; (ii) Naive Bayes is a probabilistic model based on Bayes’ Theorem and the “naive” assumption of feature statistical independence [5]; (iii) Logistic regression estimates the probability of a sample belonging to the positive class based on the log odds ratio estimation [6]; (iv) K-nearest neighbors is a non-parametric classifier underlying distance-based models. Its decision-based perspective is based on the minimization of the Euclidian distance within each target class. The k-nearest neighbor classifiers take a vote between the nearest exemplars of instance to be classified and predict the majority class.

Ensemble models, such as (v) Random Forest [7], (vi) Gradient Boosting [8], and (vii) Ada Boosting [9] learn by training a collection of individual methods combining their predictions. Random Forest is a bagging method promoting diversity among trees in the forest by learning them on bootstrap replicates of the original dataset, and finally predicting the majority voting of individual decision trees. Gradient Boosting and Ada Boosting are boosting methods, which start by assigning the same weight to all the examples; as boosting proceeds, the weights are adjusted based on the results of the prediction in order to focus the learning on the examples that were not properly addressed yet. The main differences in these two algorithms are associated with some implementation details as well as the policies used to assign the initial weights and in the implementation of the loss of function to assign penalties to the misclassified points. The AUC and accuracy values reported in the paper are the highest values obtained from one of the seven classification algorithms.

**Implementation of the framework**

The analyses workflow was implemented using Python 3 programming language [10]. Several Python modules were exploited for different purposes: scikit-learn [11] for ML algorithms, [12] for data manipulation, seaborn [11] for data visualization, and graph-tool [14] for graph analysis and visualization. The novel EmbryoMLSelection framework was registered in the Docker image, available at qbioturin/embryo_ml_workflow, in order to ensure both functional and computational reproducibility of the experiments.

**REFERENCES**

1. Pardini B. et al. A Fecal MicroRNA Signature by Small RNA Sequencing Accurately Distinguishes Colorectal Cancers: Results From a Multicenter Study, 2023, Gastroenterology in press.

2. Chicco D, Jurman G. The advantages of the Matthews correlation coefficient (MCC) over F1 score and accuracy in binary classification evaluation. BMC Genomics. 2020;21:6.

3. Huang Z. Extensions to the k-Means Algorithm for Clustering Large Data Sets with Categorical Values. Data Min Knowl Discov. 1998;2:283–304.

4. Fletcher T. Support Vector Machines Explained. 2008 [cited 2022 Aug 23]; Available from: https://www.academia.edu/22709227/Support_Vector_Machines_Explained

5. Zhang H. The Optimality of Naive Bayes. 2004 [cited 2022 Aug 23]; Available from: https://www.academia.edu/5833043/The_Optimality_of_Naive_Bayes

6. McCullagh P, Nelder JA. Generalized Linear Models, Second Edition. CRC Press; 1989.

7. Breiman L. Random Forests. Mach Learn. 2001;45:5–32.

8. Friedman JH. Greedy function approximation: A gradient boosting machine. Ann Stat. 2001;29:1189–232.

9. Van Rossum G, Drake FL. Python 3 Reference Manual. Scotts Valley, CA: CreateSpace; 2009.

10. Freund Y and Schapire R. A Decision-Theoretic Generalization of On-Line Learning and an Application to Boosting, Journal of Computer and System Sciences, 1997, 55:119-139

11. Waskom ML. seaborn: statistical data visualization. J Open Source Softw. 2021;6:3021.

12. Pedregosa, Fabian, et al. Scikit-learn: Machine learning in Python. the Journal of machine Learning research, 2011, 12: 2825-2830.

13. McKinney, W. pandas: a foundational Python library for data analysis and statistics. Python for high performance and scientific computing, 2011, 14.9: 1-9.

14. Tiago P. Peixoto, “The graph-tool python library”, figshare. (2014)

**Supplementary Table 1. Patients’ clinical characteristics and IVF outcome.** Data are shown as mean ± standard deviation or as a percentage.

|  | **Training cohort**  **(n=80 patients)** | **Validation cohort**  **(n=10 patients)** | **P value** |
| --- | --- | --- | --- |
| **Age (years)** | 35.3 ± 3.5 | 35.5 ± 3.1 | 0.97 |
| **Duration of infertility (years)** | 2.9 ± 1.5 | 2.4 ± 1.0 | 0.88 |
| **Body mass index (kg/m^2^)** | 24.2 ± 4.8 | 24.6 ± 2.6 | 0.81 |
| **Day 3 FSH (IU/l)** | 7.3 ± 3.9 | 7.3 ± 2.4 | 0.93 |
| **AMH (ng/ml)** | 5.4 ± 4.2 | 5.2 ± 3.9 | 0.84 |
| **Antral follicle count (n)** | 17.5 ± 8.3 | 16.7 ± 9.4 | 0.67 |
| **Total FSH dose (IU)** | 2137.1 ± 900 | 2121.8 ± 930 | 0.72 |
| **Ovarian sensitivity index (OSI)** | 6.8 ± 4.7 | 6.4 ± 5.1 | 0.87 |
| **Retrieved COCs (n)** | 12.1 ± 5.3 | 12.0 ± 5.6 | 0.86 |
| **Mature (MII) oocytes (%)** | 85.9 ± 14.9 | 82.8 ± 11.7 | 0.75 |
| **Fertilization rate (%)** | 73.7 ± 17.7 | 75.5 ± 16.7 | 0.78 |
| **Cleavage rate (%)** | 98.6 ± 4.9 | 98.9 ± 5.3 | 0.89 |
| **Blastulation rate/2PN (%)** | 53.8 ± 4.7 | 55.4 ± 6.1 | 0.58 |

**Supplementary Table 2. List of significant comparisons depicted in Figure 1C and described by the corresponding p-values** (Kruskal-Wallis rank sum test was used).

| **Variable** | **p-value** | **Significance Codes** |
| --- | --- | --- |
| **Age** | 9,86E-05 | *** |
| **BMI** | 0,05413 | *ns* |
| **Day 3 FSH** | 0,04158 | * |
| **Day 3 AMH** | 0,0257 | * |
| **AFC** | 0,007098 | ** |
| **Total FSH** | 0,003553 | ** |
| **Peak E2** | 0,5638 | *ns* |
| **OSI** | 0,001776 | ** |
| **Retrieved oocytes** | 0,03795 | * |
| **Mature oocytes** | 0,009774 | ** |
| **Maturation rate** | 0,514 | *ns* |
| **Fertilized oocytes** | 0,1943 | *ns* |
| **Fertilization rate** | 0,00323 | ** |
| **Cleaved embryos** | 0,2184 | *ns* |
| **Cleavage rate** | 0,9299 | *ns* |
| **IMCS** | 2,20E-16 | *** |
| **tPNf** | 1,04E-12 | *** |
| **t2** | 2,11E-13 | *** |
| **t3** | 0,0001797 | *** |
| **t4** | 5,25E-14 | *** |
| **t8** | 1,21E-13 | *** |
| **t2-tPNf** | 7,26E-05 | *** |
| **t3-t2** | 2,13E-01 | *** |
| **t4 t3** | 1,68E-09 | *** |
| **t4-t2** | 2,00E-07 | *** |
| **t8-t4** | 6,42E-08 | *** |

**Supplementary Table 3. Detailed description of the characteristics and performances of the seven classification algorithms.**

| **Dataset** | **Classifier** | **AUC** | **Accuracy** | **kappa** | **MCC** | **precision__YES** | **recall__YES** | **f1-score__YES** | **support__YES** | **precision__NO** | **recall__NO** | **f1-score__NO** | **support__NO** | **TN** | **FN** | **FP** | **TP** |
| --- | --- | --- | --- | --- | --- | --- | --- | --- | --- | --- | --- | --- | --- | --- | --- | --- | --- |
| Validation | knn | 0,726 | 0,757 | 0,437 | 0,449 | 0,721 | 0,526 | 0,606 | 29,000 | 0,771 | 0,885 | 0,824 | 52,000 | 46,032 | 5,968 | 13,749 | 15,251 |
| Validation | log_reg | 0,840 | 0,800 | 0,532 | 0,553 | 0,829 | 0,559 | 0,667 | 29,000 | 0,792 | 0,935 | 0,857 | 52,000 | 48,607 | 3,393 | 12,794 | 16,206 |
| Validation | r_forest | 0,810 | 0,766 | 0,459 | 0,471 | 0,740 | 0,536 | 0,622 | 29,000 | 0,776 | 0,894 | 0,831 | 52,000 | 46,512 | 5,488 | 13,443 | 15,557 |
| Validation | g_boost | 0,805 | 0,765 | 0,461 | 0,470 | 0,727 | 0,552 | 0,628 | 29,000 | 0,780 | 0,884 | 0,829 | 52,000 | 45,985 | 6,015 | 12,983 | 16,017 |
| Validation | svm | 0,715 | 0,765 | 0,452 | 0,468 | 0,750 | 0,517 | 0,612 | 29,000 | 0,770 | 0,904 | 0,832 | 52,000 | 47,000 | 5,000 | 14,000 | 15,000 |
| Validation | gauss_nb | 0,834 | 0,775 | 0,511 | 0,511 | 0,688 | 0,683 | 0,685 | 29,000 | 0,824 | 0,827 | 0,825 | 52,000 | 43,000 | 9,000 | 9,194 | 19,806 |
| Validation | adaboost | 0,842 | 0,814 | 0,566 | 0,586 | 0,847 | 0,587 | 0,693 | 29,000 | 0,803 | 0,941 | 0,867 | 52,000 | 48,925 | 3,075 | 11,980 | 17,020 |

**Supplementary Figure 1.** ***EmbryoMLSelection* framework.** The framework is composed of four modules: Feature Selection, Rules extraction, Rules selection and Rules evaluation. Rules can be visualized by a graph structure (correlation graph) in order to help the inspection of their association with the outcome and among each other**.** The output obtained is reported highlighted in green for each module.

**Supplementary Figure 2.** (A) Correlation graph of the *extracted* rules obtained considering all variables; vertices represent the rules and arcs are reported only for correlation value >0.8 (computed by Matthews Correlation Coefficient (MCC). (B) Line plot representing the ability of different combinations of classifiers to classify expanded or not expanded blastocyst stage. Each dot corresponds to the AUC computed using a different number of rules in input.

Supplementary Figure 3. (A) Correlation graph of the extracted rules obtained considering only the embryo-variables; vertices represent the rules and arcs are reported only for correlation value >0.8 (computed by Matthews Correlation Coefficient (MCC). (B) Line plot representing the ability of different combinations of classifiers to classify expanded or not expanded blastocyst stage. Each dot corresponds to the AUC computed using a different number of rules in input. The light blue graph provides a hierarchical representation of the communities composed of nodes highlighted in the large graph with the same color (squared nodes), allowing the visualization of interconnection among communities. This rapresentation is generated using graph-tool Python library (https://graph-tool.skewed.de/) with the minimize_nested_blockmodel_dl() function.It relies on the Nested Stochastic Block Model, a generative model (for community detection) with parameters estimated using a Bayesian probabilistic approach.
